# Secondary failure of platelet recovery after allogeneic hematopoietic stem cell transplantation in pediatric acquired aplastic anemia: Incidence, risk factors, and clinical outcomes

**DOI:** 10.46989/001c.171012

**Published:** 2026-09-30

**Authors:** Yue Shang, Yuanyuan Ren, Lipeng Liu, Xin Wang, Chaoqian Jiang, Xia Chen, Yang Wan, Aoli Zhang, Xiaoyan Zhang, Fang Liu, Li Zhang, Yumei Chen, Yao Zou, Xiaojuan Chen, Wenyu Yang, Xiaofan Zhu, Ye Guo

**Affiliations:** 1 Department of Pediatric Hematology, State Key Laboratory of Experimental Hematology, National Clinical Research Center for Blood Diseases, Haihe Laboratory of Cell Ecosystem, Institute of Hematology & Blood Diseases Hospital, Chinese Academy of Medical Sciences & Peking Union Medical College, Tianjin 300020, China; 2 Tianjin Institute of Health Science, Tianjin 301600, China

**Keywords:** Hematopoietic stem cell transplantation (HSCT), child, Secondary failure of platelet recovery (SFPR)

## Abstract

Secondary failure of platelet recovery (SFPR) poses a significant risk for life-threatening complications following hematopoietic stem cell transplantation (HSCT). However, the incidence, clinical outcomes, and risk factors associated with SFPR in the context of allogeneic HSCT (allo-HSCT) for pediatric patients with acquired aplastic anemia (AA) remain unclear. We conducted a retrospective review of 86 consecutive acquired AA children who underwent allo-HSCT at our center from December 2017 to November 2023. The 1-year cumulative incidence of SFPR was found to be 11.6% (95% confidence interval [CI]: 6.2%-21.1%). The median time to SFPR was 2 months (range: 1-5 months) post-transplantation. To clarify the risk factors for SFPR, 9 SFPR cases and 69 non-SFPR cases were further analyzed. Multivariable analyses revealed that age, pre-transplantation body mass index (BMI) and a history of grade Ⅲ-Ⅳ acute graft-versus-host disease (aGVHD) were the most significant predictors for SFPR post-transplantation (p < 0.05). Additionally, we found that the 2-year overall survival rate was significantly lower in children with SFPR compared to those without (74.1% vs. 96.7%; p < 0.005). Ultimately, 4 patients achieved full platelet count recovery, with 2 patients becoming transfusion-independent. However, 1 patient still requires periodic platelet transfusions, and 2 patients unfortunately died.Among the 6 patients who received thrombopoietin receptor agonist therapy, an overall response rate of 66.6% was observed, suggesting the potential efficacy of this treatment approach in managing SFPR. This study underscores the need for vigilant monitoring of platelet recovery in pediatric HSCT recipients and highlights the need for further research into the etiology of SFPR to improve clinical outcomes.

## Introduction

Survival after hematopoietic stem cell transplantation (HSCT) for acquired aplastic anemia (AA) has improved significantly in recent decades.^1,2^ Sustained hematopoietic recovery is pivotal to the success of HSCT in AA. However, clinicians have noted that even when patients achieve initial hematopoietic reconstitution and retain complete donor-derived hematopoietic cells, they may subsequently develop refractory thrombocytopenia.^3,4^ This phenomenon, known as secondary failure of platelet recovery (SFPR), affects 12-26% of patients post-transplantation according to previous reports.^5–7^ Patients with SFPR lose their initial platelet reconstitution, which leads to increased risks of severe bleeding events and other life-threatening complications, or requirement for transfusions after transplantation. Current therapeutic strategies for SFPR are constrained, and the associated prognosis remains poor.

Several risk factors have been associated with SFPR after allogeneic HSCT, including transplants from unrelated donors, graft-versus-host disease (GVHD) prophylaxis regimens, severe acute GVHD (aGVHD), conditioning involving busulfan, cyclophosphamide and total body irradiation, stem cell dosage, and infections—particularly cytomegalovirus (CMV) reactivation.^5,6^ However, data on SFPR after allogeneic HSCT (allo-HSCT) in pediatric AA patients are still limited.

This study aims to investigate the incidence, clinical management, outcomes, and risk factors associated with SFPR in pediatric patients with AA post-HSCT.

## Materials and Methods

### Patient Selection

In this retrospective analysis, we reviewed a consecutive series of 86 acquired AA children who underwent allo-HSCT as their first HSCT at the Institute of Hematology and Blood Diseases Hospital in Tianjin, China, from December 2017 to November 2023. All pediatric patients’ guardians gave informed consent before participation in this study. Patients who developed graft failure after allo-HSCT (n=5) or died of any cause within 30 days post-transplantation without hematopoietic reconstitution (n=3) were excluded from further analysis. Consequently, the remaining cohort, comprising 78 patients, was utilized to estimate the cumulative incidence of secondary failure of platelet recovery (SFPR).

### Definitions

The timeline for SFPR was calculated from the date of HSCT to the date of clinical diagnosis. SFPR was defined as the first of seven consecutive days of platelet counts <20 ×10^9^/L or a requirement of platelet transfusions within 7 days after primary platelet recovery was achieved. Patients who had graft rejection or disease relapse within 30 days after the onset of SFPR were regarded as those without SFPR, in accordance with previous studies.^7^ Neutrophil engraftment was identified as the first of three consecutive days with an absolute neutrophil count exceeding 0.5 ×10^9^/L, while platelet engraftment was defined as the first of seven consecutive days with an unsupported platelet count above 20 ×10^9^/L. Cytomegalovirus (CMV) and Epstein-Barr virus (EBV) DNAemia was defined as the presence of >1000 DNA copies/mL in blood by quantitative polymerase chain reaction (PCR) assays.^8,9^ A human leukocyte antigen (HLA) mismatch was identified as having at least one antigen discrepancy between the donor and recipient. Both aGVHD and chronic GVHD (cGVHD) were diagnosed and graded according to established criteria.^10^ Body mass index (BMI) was categorized according to World Health Organization BMI-for-age standards. Overall survival (OS) was calculated as the period from the date of transplantation to the date of death from any cause or the last follow-up point.

### Statistical Analysis

The last follow-up for all survivors was conducted on February 25th, 2024. All clinical data were analyzed utilizing R software (version 4.4.1). Continuous variables were summarized as median (range) and compared using the Mann-Whitney U test, while categorical variables were presented as counts and percentages and compared using the chi-square test or Fisher’s exact test. Univariable analysis was performed based on logistic regression models, and potential risk factors (p-value <0.10) were further analyzed in multivariable analysis. The final model was determined using stepwise multiple logistic regression with the “MASS” package.^11^ Furthermore, the Kaplan-Meier method was employed to estimate survival curves. All statistical tests were two-tailed, and a p-value <0.05 was considered to indicate statistical significance. The cumulative incidence rate (CIR) of SFPR was calculated using Gray’s method, considering death without SFPR as a competing event.

## Results

### Characteristics of patients with SFPR or non-SFPR

A total of 78 pediatric patients with acquired AA who underwent allo-HSCT were included. The median age was 8 (range, 2-17) years, and 60.2% were male. Pre-transplant, 52.6% were diagnosed with very severe AA (VSAA). The donor types for HSCT were as follows: 59.0% received grafts from haploidentical donors (Haplo), 28.2% from matched sibling donors (MSD), and 12.8% from unrelated umbilical cord blood (UCB). Additional general characteristics of the patients are presented in Table 1.

**Table 1.** Demographic and Clinical Characteristics of Patients with SFPR or non-SFPR.

*Characteristic*	*Total* *n=78*	*SFPR* *n=9*	*non-SFPR* *n=69*
Age, years, median (range)	8 (2-17)	10 (6-17)	8 (2-17)
Male, n (%)	47 (60.2)	7 (77.8)	40 (58.0)
BMI, n (%) Normal Overweight Obesity Thinness	48 (61.5) 14 (17.9) 12 (15.4) 4 (5.1)	2 (22.2) 3 (33.3) 2 (22.2) 2 (22.2)	46 (66.7) 11 (15.9) 10 (14.5) 2 (2.9)
VSAA, n (%)	41 (52.6)	5 (55.6)	36 (52.2)
AA course, months, median (range)	4 (1-105)	12(2-56)	4 (1-105)
GVHD prophylaxis, n (%) CSA based TAC based	70 (89.7) 8 (10.3)	8 (88.9) 1 (11.1)	62 (89.9) 7 (10.1)
SF level, ng/mL, median (range)	335.1 (29.8-1667.0)	303.2 (102.5-499.4)	337.8 (29.8-1667.0)
Donor age, years, median (range)	28 (0-55)	37 (8-55)	24 (0-53)
Related Donor source, n (%)	68 (87.2)	9 (100.0)	59 (85.5)
Stem cell source, n (%) BM+PB PB BM CB	21 (26.9) 46 (59.0) 1 (1.3) 10 (12.8)	3 (33.3) 6 (66.7) 0 (0.0) 0 (0.0)	18 (26.1) 40 (58.0) 1 (1.4) 10 (14.5)
Donor-recipient sex match, n (%)	42 (53.9)	7 (77.8)	35 (50.7)
HLA match 9~10/10, n (%)	35 (44.9)	2 (22.2)	33 (47.9)
ABO blood group match, n (%)	39 (50.0)	5 (55.6)	34 (49.3)
Reduced intensity Conditioning regimen, n (%)	60 (76.9)	9 (100.0)	51 (73.9)
Graft characteristics MNCs (×108/kg), median (range) CD34+cells(×106/kg), median(range)	10.0 (0.3-21.3) 2.6 (0.1-11.7)	10.7 (10.0-15.0) 3.3 (1.2-5.0)	10.0 (0.3-21.3) 2.5 (0.1-11.7)
Donor type, n (%) Haplo MSD UCB	46 (59.0) 22 (28.2) 10 (12.8)	7 (77.8) 2 (22.2) 0 (0.0)	39 (56.5) 20 (29.0) 10 (14.5)

VSAA: very severe aplastic anemia; CSA: cyclosporin; TAC: tacrolimus; SF: serum ferritin；BM：bone marrow；PB：peripheral blood；MNCs：total number of mononuclear cells transfused; Haplo: haploidentical donors; MSD: matched sibling donors; UCB: unrelated umbilical cord blood.

### Incidence of SFPR and other clinical events before SFPR onset

The median time to SFPR was 2 (range, 1-5) months post-transplantation (Supplemental Figure 1). The 1-year CIR of SFPR was 11.6% (95% CI: 6.2%-21.1%). Among the SFPR cohort, the median times to neutrophil and platelet engraftment were both 14 days post-transplantation. Four patients (44.4%) had developed grades III-IV aGVHD prior to the onset of SFPR. Additionally, 3 patients (33.3%) experienced CMV reactivation, transplantation-associated thrombotic microangiopathy (TA-TMA), and hemorrhagic cystitis (HC), respectively. Furthermore, 2 individuals had evidence of EBV activation and early bloodstream infection, respectively. Full cohort characteristics are detailed in Table 2.

**Table 2.** Clinical events before SFPR onset after allo-HSCT.

*Events*	*SFPR* *n=9*	*non-SFPR* *n=69*
Neutrophil engraftment,day, median (range)	14（11-17）	13 (10-27)
Platelet engraftment, day, median (range)	14（12-30）	15 (8-124)
Ⅲ-Ⅳ aGVHD, n (%)	4 (44.4)	12 (17.4)
EBV DNAemia, n (%)	2 (22.2)	8 (11.6)
CMV DNAemia, n (%)	3 (33.3)	27 (39.1)
TA-TMA, n (%)	3 (33.3)	7 (10.1)
Early bloodstream infections, n (%)	2 (22.2)	8 (11.6)
HC, n (%)	3 (33.3)	24 (34.8)

Ⅲ-Ⅳ aGVHD: grades III-IV acute graft-versus-host disease; EBV: Epstein-Barr virus; CMV: Cytomegalovirus; TA-TMA: transplantation-associated thrombotic microangiopathy; HC: hemorrhagic cystitis.

### Risk Factors for SFPR

We subsequently explored whether patients’ characteristics, HSCT program, and early post-transplantation complications could predict the incidence of SFPR. The results of the univariate logistic regression analysis for SFPR are presented in Supplementary Table 1. Subsequently, we selected indicators with P-values < 0.1 for inclusion in the multivariate analysis. On the latter, age, BMI and Ⅲ-Ⅳ aGVHD (p-value <0.05) were identified as the most significant predictors for SFPR (Table 3). These findings suggest that special attention should be given to patients with abnormal BMI and those who have experienced severe aGVHD, as they may be at higher risk for the development of SFPR.

**Table 3.** Multivariate analysis for factors associated with SFPR in children with aplastic anemia after transplantation.

*Variables*	*Adjusted OR for all patients (95% CI)*	*P value*
Age(year)	1.431 (1.087-2.049)	0.023
BMI (kg /m^2^) Normal Overweight Obesity Thinness	Ref 14.069 (1.378-276.396) 16.793 (1.103-486.145) 51.813(2.646-2107.124)	0.040 0.055 0.015
Ⅲ-Ⅳ aGVHD	24.585 (2.557-519.787)	0.014

BMI: Body mass index; Ⅲ-Ⅳ aGVHD: grades III-IV acute graft-versus-host disease

### Managements and outcomes of SFPR

Treatment strategies for the 9 patients with SFPR, included platelet transfusions, thrombopoietin receptor agonists (TPO-RAs), recombinant human thrombopoietin (rhTPO), and glucocorticosteroid (Shown in Table 4). Ultimately, 4 patients (patients 3, 4, 5, and 7) achieved full platelet count recovery. In 2 cases (patients 2 and 9), the interventions resulted in a state of transfusion independence, with absolute platelet values consistently above 20 × 10^9^L, although not returning to the normal range. Patient 1 did not experience recovery and remained dependent on platelet transfusions. Two patients (nos. 6 and 8) succumbed to severe pneumonia. The treatment outcomes are shown in Supplemental Figure 1.

**Table 4.** Demographic and Clinical Profiles for SFPR Patients

*Patients*	*Age (Years)*	*Gender*	*Disease* *type*	*Donor* *Type*	*Neutrophil and Platelet Engraftment* *(Days)*	*SFPR* *Onset Time* *(Days)*	*Transplant-related complications*	*treatments*	*outcome*
1	8	Male	VSAA	MSD	14,13	60	None	TPO-RAs, MSC	Transfusion- dependent
2	10	Male	NSAA	Haplo	11,12	45	HC, blood stream infection, Ⅱ aGVHD	TPO-RAs, glucocorticosteroid	Transfusion- independent
3	6	Male	SAA	Haplo	11,12	35	TA-TMA, Ⅳ aGVHD	TPO-RAs, glucocorticosteroid, MSC, IVIG	recovery
4	11	Male	VSAA	Haplo	17,21	150	CMV DNAemia	TPO-RAs	recovery
5	11	Male	VSAA	MSD	12,12	55	CMV DNAemia, Ⅲ aGVHD	Glucocorticosteroid, rhTPO	recovery
6	8	Male	NSAA	Haplo	11,14	22	blood stream infection	Immunosuppressant dose reduction	Dead
7	10	Female	VSAA	Haplo	15,16	90	CMV DNAemia, EBV DNAemia, HC,TA-TMA, Ⅲ aGVHD	TPO-RAs	recovery
8	11	Male	VSAA	Haplo	14,22	71	EBV DNAemia, TA-TMA, Ⅳ aGVHD	TPO-RAs, glucocorticosteroid	Dead
9	17	Male	SAA	Haplo	17,30	60	HC, I aGVHD	Immunosuppressant dose reduction, glucocorticosteroid	Transfusion- independent

SAA: severe aplastic anemia; NSAA: non-severe aplastic anemia; VSAA: very severe aplastic anemia; Haplo: haploidentical donors; MSD: matched sibling donors; CMV: cytomegalovirus; EBV: Epstein-Barr virus; TA-TMA: transplantation-associated thrombotic microangiopathy; HC：hemorrhagic cystitis; IVIG: intravenous immunoglobulin; TPO-RAs: thrombopoietin receptor agonists; rhTPO: recombinant human thrombopoietin; MSC: mesenchymal stem cell.

Among the five patients treated with avatrombopag and the one treated with eltrombopag, three regained normal platelet counts, and one became independent of platelet transfusions, yielding an overall response rate of 66.6%.

### Impact of SFPR on OS

With a median follow-up period of 32 (range, 0.8 to 75.1) months), the 2-year OS rate for the entire cohort was 94.3% (95% CI 89.1-99.9). Notably, a significant difference in OS was observed between patients with and without SFPR (74.1% versus 96.7%; p < 0.005) (Figure 1).

**Figure 1.**
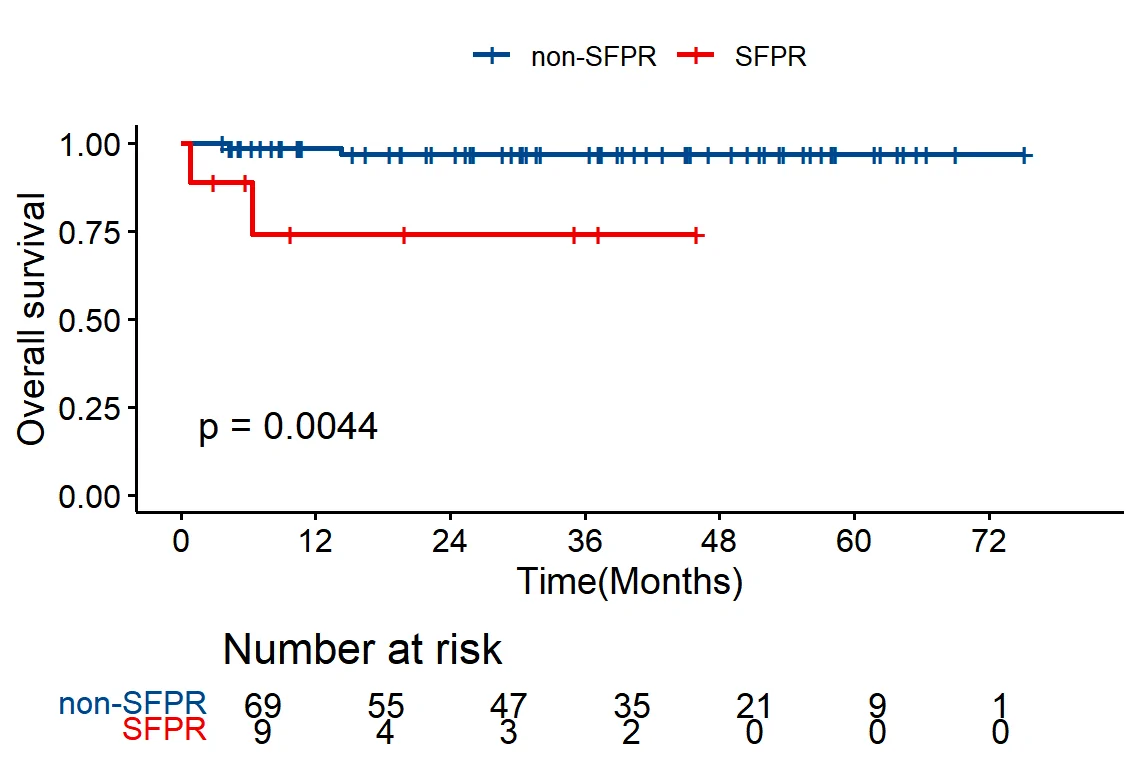
The overall survival of pediatric patients with SFPR.

## Discussion

Acquired AA in children is a severe hematological disorder that seriously not only endangers children’s physical and mental health, but also imposes a heavy economic burden on families and society, while consuming substantial public health resources. In the case of SAA/VSAA and transfusion-dependent AA, allo-HSCT is a key therapeutic option. In our studies, the 2-year OS rate for the whole cohort was 94.3% (95% CI 89.1-99.9), which indicates a marked improvement in prognosis for pediatric patients with AA.

SFPR, as an important complication following HSCT, is associated with life-threatening hemorrhage, negatively impacting the quality of survival in pediatric patients. Previous research has mostly focused on adult allogeneic transplants or autologous transplants,^6^ with only a few reports addressing SFPR in the context of pediatric allo-HSCT. In our study, 11.6% (9/78) of the children developed SFPR (95% CI: 6.2%-21.1%) within 1-5 months post-transplantation. This incidence appears to be slightly lower than that observed in adults, which ranges from 12% to 26%.^5–7^ Although older age in our cohort seems to correlate with a higher risk of SFPR, this observation does not firmly establish that age plays a significant role in the incidence of SFPR, given the limited studies comparing different age groups.

Importantly, the etiology of SFPR is not yet fully understood. Some specialists^12^ have suggested an association between SFPR and aGVHD. Research has shown that patients with SFPR/aGVHD have significantly higher levels of inflammatory cytokines IL-2R and TNF-R1, indicating a dysregulated immune response. Additionally, pathways critical to hematopoiesis and immune responses are underexpressed in CD34+ cells isolated from SFPR/aGVHD patients, suggesting a direct impact on hematopoietic function. Our multifactorial analyses seem to support these findings, highlighting the need for further investigation into the relationship between SFPR and aGVHD. Interestingly, we identified abnormal BMI as a potential risk factor for the development of SFPR. Previous research has reported^13,14^ that both obesity and low BMI can increase the risk of aGVHD after post-transplantation. In our study, 7/9 patients with SFPR had abnormal BMIs (3 cases of overweight, 2 cases of obesity, and 2 cases of thinness), with only 2 cases simultaneously developing grades Ⅲ-Ⅳ aGVHD. This suggests a complex interplay between BMI, aGVHD, and SFPR that warrants further exploration. As for CMV reactivation, contrary to a previous report by Zhao et al.,^15^ which suggested a higher incidence of CMV reactivation in SFPR patients, our findings indicate that out of 9 patients in the SFPR group, only 3 had CMV with an average viral load of 5707.67 CMV DNA copies/mL in the serum. This was lower than the average of 8095.67 CMV DNA copies/mL observed in 27 non-SFPR patients with CMV reactivation, suggesting that the role of CMV in SFPR may be more complex than previously thought.

In recent years, TPO-RA is becoming increasingly important in the treatment of patients who develop thrombocytopenia due to various causes.^3,16–18^ Among the 6/9 patients with SFPR, who received TPO-RA monotherapy or combination therapy, the overall response rate was 66.6%. As reported,^19^ rhTPO has also demonstrated efficacy in the treatment of SFPR. In our cohort, platelet counts gradually returned to normal in one patient who received rhTPO and methylprednisolone. However, restricted by the limited number of cases, we are unable to compare the efficacy of different treatment regimens. Further in-depth mechanistic studies are needed to guide us to more efficient clinical treatments.

The present study encompasses several limitations. The single-center retrospective design and limited cohort size (particularly the low SFPR incidence yielding few positive cases) may compromise statistical power and increase the risk of model overfitting. Multivariable regression analyses were potentially limited by an inadequate events-per-variable (EPV) ratio, raising concerns about parameter estimate stability. Finally, the absence of external validation restricts generalization of findings. While univariate analyses were conducted to mitigate these constraints, confirmation through larger prospective cohorts remains essential. Future studies with larger numbers of patients are needed to validate our findings and to explore the associations identified in this preliminary analysis.

In summary, our findings revealed that 11.6% of pediatric patients who underwent transplantation developed SFPR which was not due to relapse or graft rejection. We identified abnormal BMI and severe aGVHD were significant risk factors associated with SFPR. Additionally, SFPR had a significant adverse effect on OS. Therefore, novel approaches to determine the underlying etiology of SFPR will be required to improve the outcome of patients with SFPR.

### Authors’ Contribution - CRediT

*Conceptualization*: Ye Guo; Methodology: Yuanyuan Ren, Lipeng Liu; *Formal analysis and investigation*: Yue Shang; *Writing - original draft preparation*: Yue Shang; *Writing - review and editing*: Xin Wang, Chaoqian Jiang, Xia Chen, Yang Wan, Aoli Zhang, Xiaoyan Zhang, Fang Liu, Li Zhang, Yumei Chen, Yao Zou, Xiaojuan Chen, Wenyu Yang; *Funding acquisition*: Ye Guo ; *Resources*: Xiaofan Zhu, Ye Guo; *Supervision*: Ye Guo

### Competing of Interest – COPE

No competing interests were disclosed.

### Ethical Conduct Approval – Helsinki – IACUC

The study design and methods complied with the Declaration of Helsinki and were approved by the Ethics Committee and Institutional Review Board of the Institute of Hematology and Blood Diseases Hospital, Chinese Academy of Medical Sciences & Peking Union Medical College.

### Informed Consent Statement

All authors and institutions have confirmed this manuscript for publication.

### Data Availability Statement

All are available upon reasonable request.

## Figures and Tables

**Supplemental Figure 1.**
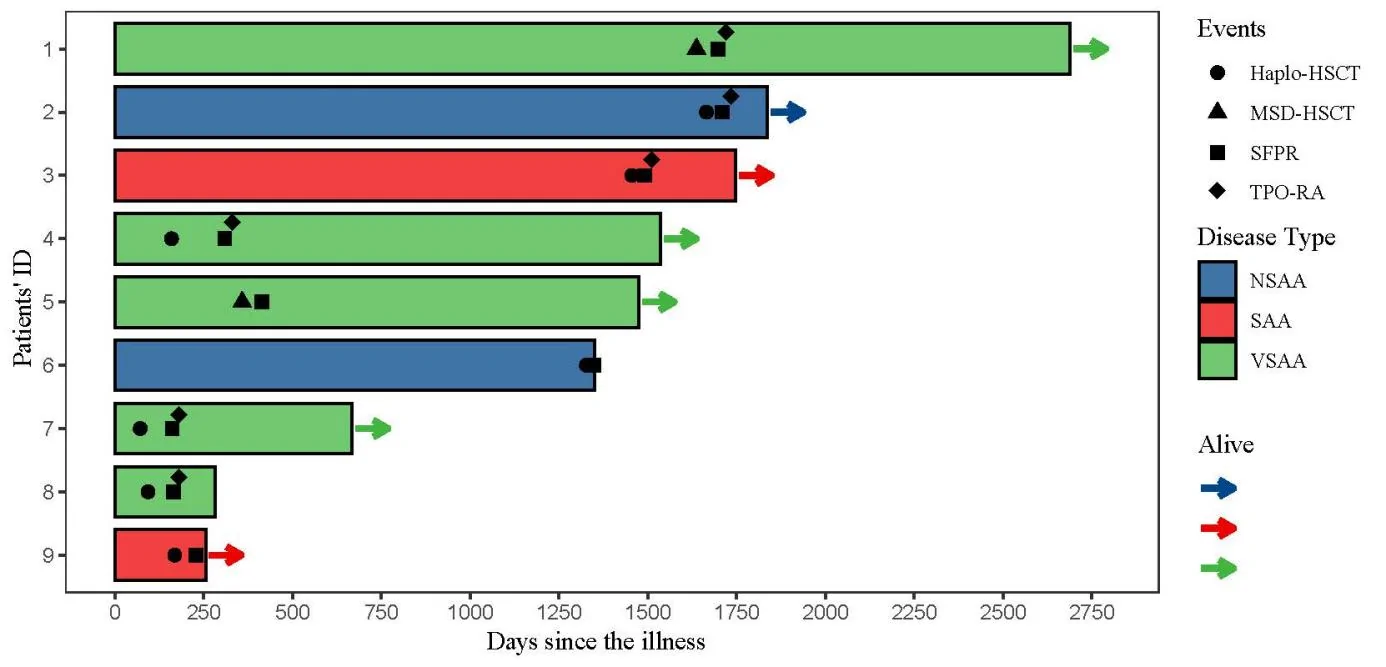
The timeline of pediatric patients with SFPR.

## Supplemental material

**Supplemental Table 1.** Univariate analysis for factors associated with SFPR in children with aplastic anemia after transplantation.

	*Univariate logistic analysis*	
	*OR (95%CI)*	*P value*
Gender Male Female	Ref 2.538(0.564-17.873)	0.267
Age (year)	1.234(1.004-1.548)	0.052
BMI Normal Overweight Obesity Thinness	Ref 6.273(0.935-52.243) 4.600(0.504-42.309) 23.000(1.993-319.918)	0.059 0.150 0.011
Time from diagnosis to transplantation (month)	1.000(0.999-1.001)	0.301
Disease type		
SAA/NSAA	Ref	
VSAA	1.146(0.280-4.973)	0.849
Conditioning regimen intensity		
MAC	Ref	
RIC	2.002×107(0.000-NA)	0.992
Conditioning regimen scheme		
Non-BU	Ref	
BU-based	6.736×107(0.000-NA)	0.993
Donor and recipient ABO blood		
Not matched	Ref	
matched	0.956(0.180-7.253)	0.960
Donor and recipient sex		
Not matched	Ref	
matched	3.400(0.758-23.925)	0.144
HLA match		
5-8/10	Ref	
9-10/10	0. 312(0.044-1.399)	0.164
Relative donor No Yes	Ref 1.764×107(0.000-NA)	0.994
Total number of mononuclear cells transfused (×108/L)	1.118(0.947 -1.357)	0.218
Total number of CD34+ cell transfused (×106/L)	1.045(0.721-1.410)	0.791
Pretransplant ferritin (ng/ml)	0. 993(0.962-1.025)	0.677
Early bloodstream infections No Yes	Ref 2.179(0.291-11.098)	0.379
CMV DNAemia No Yes	Ref 0.778(0.154-3.214)	0.737
EBV DNAemia No Yes	Ref 3.813(0.701-17.838)	0.095
Ⅲ-Ⅳ aGVHD No Yes	Ref 3.800(0.837-16.550)	0.072
Hemorrhagic cystitis No Yes	Ref 0.938(0.185-3.893)	0.932
TA-TMA No Yes	Ref 4.439(0.802-21.308)	0.067

SAA: severe aplastic anemia; NSAA: non-severe aplastic anemia; VSAA: very severe aplastic anemia; MAC: myeloablative conditioning; RIC: reduced intensity conditioning; BU: busulfan; CMV: cytomegalovirus; EBV: Epstein-Barr virus; TA-TMA: transplantation-associated thrombotic microangiopathy; BMI: Body mass index; III-IV aGVHD: grades III-IV acute graft-versus-host disease
